# Attractive force-driven superhardening of graphene membranes as a pin-point breaking of continuum mechanics

**DOI:** 10.1038/srep46083

**Published:** 2017-04-18

**Authors:** Makoto Ashino, Roland Wiesendanger

**Affiliations:** 1 Faculty of Engineering, Kanazawa Institute of Technology, Nonoichi, 921-8501, Japan; 2 Institute of Applied Physics, University of Hamburg, Hamburg, 20355, Germany

## Abstract

Bending at the nanometre scale can substantially modify the mechanical, chemical and electronic properties of graphene membranes. The subsequent response of chemical bonds leads to deviations from plate idealisation in continuum mechanics. However, those phenomena have thus far been investigated exclusively by measuring the electronic properties of graphene deformed by compressing and stretching with local-probe techniques. Here, we report that the interatomic-attractive forces applied on the convexly-curved graphene by the probe tip give rise to a pin-point breaking of the plate idealisation in the continuum mechanics, facilitating atomically-localised enhancements in its chemical reactivity and mechanical strength. Thorough characterisations were conducted by atomic force microscopy and force field spectroscopy on hollow nanotubes, rolled-up graphene, with different diameters. Their topmost parts supplied well-defined curvatures of the convex graphene. We found that a significant enhancement in the out-of-plane Young’s modulus from 13 to 163 GPa, “superhardening”, was realised with the nonlinear transition of bond configurations. Our findings provide a fundamental understanding of the relationships between the structure of atomistic membranes and the dynamic behaviour of approaching exterior atoms or molecules and their subsequent interplay with chemical and mechanical properties. Thus, these results encourage the application of such membranes in functionally-controllable materials or devices.

Graphene is a single atomic layer of ***sp***^**2**^-bonded carbon atoms arranged in a two-dimensional (2D) honeycomb lattice and a basic building block for graphitic materials of all other dimensionalities, such as 0D fullerenes (spherical graphene), 1D nanotubes (rolled-up graphene), and 3D graphite (stacked graphene)^1^,^2^,^3^,^4^,^5^. Owing to the unique combination of an extremely small out-of-plane stiffness with a high in-plane modulus (~1000 GPa) and tensile strength (~100 GPa), the behaviour of curved graphene is of fundamental importance for studying graphene-based nanostructures ranging from 0D to 3D and for their application in a variety of devices^4^,^6^,^7^,^8^,^9^,^10^. The bending properties not only control the morphology of graphene under external stimuli^11^,^12^,^13^,^14^ but are also related to its electronic, magnetic, and chemical properties^1^,^2^,^3^,^6^,^15^,^16^,^17^,^18^,^19^,^20^. The carbon atoms located within the plane of graphene are chemically inert due to **π**-conjugation, whereas the curved **π**-conjugation in the carbon networks of curved graphene has not only **π**-character but also substantial ***σ***-character (i.e., **π**-***σ*** re-hybridisation)^21^,^22^. According to the “**π**-orbital axis vector” (POAV) theory, carbon atoms that reside on highly curved surfaces exhibit increased chemical potential due to diminished electronic delocalisation^22^,^23^,^24^,^25^,^26^,^27^. When the local curvature is on the nanometre scale, the electronic structure is substantially modified by altering the **π**-orbital energy and modifying the nearest-neighbour hopping integrals, which can induce a local shift in the electrochemical potential^28^ and give rise to large pseudomagnetic fields^29^.

Regarding the characterisation of the mechanical properties of curved graphene (or hollow nanotubes), there are always concerns about the applicability of existing continuum mechanics theories^6^,^30^,^31^,^32^. Although only atomically thick, graphene membranes under bending can be still described by these theories^33^,^34^. However, they usually require slowly varying, harmonic deformation conditions. These conditions are violated in realistic situations, such as sub-nanometre ripples or out-of-plane displacements of individual atoms in the carbon networks, which may be beyond first-order continuum elasticity^30^,^31^,^35^. This calls for a fundamental study of the geometry of atomistic membranes and their subsequent coupling to electronic degrees of freedom, down to unavoidable atomic-scale fluctuations^11^,^12^,^35^. The discrete geometry is relevant for addressing spin diffusion in rippled graphene^35^,^36^ as well as for understanding the chemical properties of nonplanar 2D crystals^37^, and it may even be important for the strain engineering of 2D crystals with topological defects^35^.

Under a pure bending distortion of single-layered graphene, likely caused by being rolled-up around an arbitrary axis into a hollow nanotube (Fig. 1a) from a plane (Fig. 1b), each carbon atom and its three nearest neighbours are no longer planar but are instead located in the corners of a pyramid. This pyramidalization is accounted for using the POAV construction, as indicated by arrows in the insets of Fig. 1a,b 23,24,25,26,27,30. The geometrical tilting of ***σ***_***i***_-bonds (*i* = 1, 2, 3) by an angle **θ**_**p**_ (Fig. 1a) is accomplished in POAV by introducing a degree of ***p***_***z***_ atomic orbital mixing into the ***σ***_***i***_ framework. Note that to the first order in curvature (**1**/**R**), the three tilting angles as well as the bond lengths are common^38^,^39^. Remarkably, the pyramidalization angle **θ**_**p**_ is sufficient for describing the curvature-induced shift in ***sp***^**2**^ hybridisation^30^ and is useful for gauging the reactivity of the carbon atom sites of the curved graphene^21^.

## 3D topographies and force fields on convexly curved graphene

**Figure 1:**
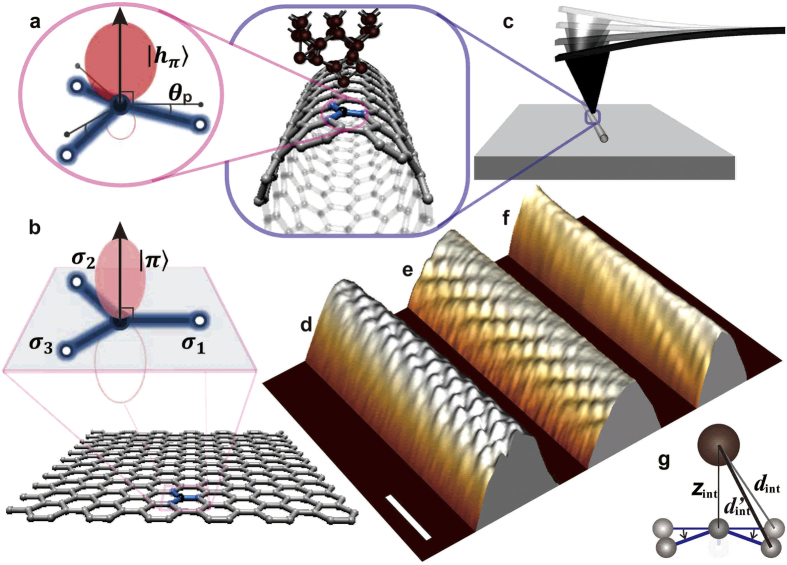
Atomic-scale AFM imaging of convexly curved graphene with different curvatures. (**a–c**) Diagrams and schematic illustrations of the *h*_*π*_-orbital in transition from *sp*^2^ to *sp*^3^ bonding configurations for the rolled-up form of graphene, i.e., nanotube, and silicon AFM tip apex (**a**), of the n-orbital in the planar graphene (**b**), and of dynamic-mode AFM imaging of the nanotube isolated on a planar substrate (**c**). The n-orbital axis vector (POAV) is indicated by an arrow for a conjugated carbon atom (●) bonded with the nearest neighbours (○) by the *σ*_*i*_-bond (*i* = 1, 2 and 3). The pyramidalization angle θ_p_ is defined by the angle between the POAV and *σ*_*i*_-bond minus 90°, as depicted in (**a**). (**d–f**) Atomic-resolution AFM topographies of the nanotubes with different radii of curvature in 3D views^58^. The scale bar is 1 nm. The nanotubes were sparsely deposited on an atomically flat substrate. Atomically resolved AFM topographies were obtained in ultrahigh vacuum (<10^−8^ Pa) at a low temperature (<15 K) under frequency modulation feedback control to maintain a constant frequency shift Δ*f* = −98.2 Hz (**d**), −63.8 Hz (**e**), and −53.3 Hz (**f**) of the cantilever resonant oscillation (*f*_o_ ≅ 159 kHz) such that a constant attractive force would be continuously acting on the tip apex over the sample surfaces. The original radii R_o_ = 8.1 Å (**d**), 7.5 Å (**e**), and 6.5 Å (**f**) were determined, respectively, with a standard deviation of 3.8***%*** (±0.25 Å) at the maximum by comparing the overall heights in their AFM topographies with those of the standard nanotube with a well-defined radius R_o_ = 6.9 ± 0.1 Å (see text). (**g**) Diagram of change in the interatomic distance *d*_int_ from the tip-apex atom to the nearest neighbour of the closest carbon atom with and without bending. The distance to the closest carbon atom is denoted by *z*_int_.

The nanotubes used in our study were single-walled carbon nanotubes (SWNTs); their original radii **R**_**o**_ ranged from 6.3 to 9.2Å^40^. The individual **R**_**o**_ values were determined by comparing the overall heights in their topographies with those for the well-defined standard of **R**_**o**_ = 6.9 ± 0.1 Å^41^. The validity is based on the finding that the overall heights are linearly correlated to **R**_**o**_ as long as <10 Å. The **R**_**o**_ values obtained in this way show good agreement with those obtained in advance using radial breathing modes in Raman spectroscopy^40^. The topographies, representing slender and convexly curved features, as shown in Fig. 1d–f, were measured over the individually isolated nanotubes on the same substrate by atomic force microscopy (AFM)^42^ with the same silicon (**Si**) tip with an atomically sharp apex. Atomically resolved topographies enable us to determine the chiral indices (**n, m**) that are utilised to confirm the accuracy of the evaluated **R**_**o**_ values^43^. The **R**_***o***_values of the nanotubes in Fig. 1d–f were found to be 8.1 Å, 7.5 Å, and 6.5 Å, respectively, with a standard deviation of 3.8%(±0.25 Å) at the maximum.

The atomically resolved topographies obtained with the same tip represent characteristic features unique to the respective nanotubes with different radii (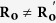), as three-dimensionally demonstrated in Fig. 1d–f. The upper part of the slender and convexly curved features in Fig. 1d, corresponding to the topmost area of the **R**_**o**_ = 8.1 Å nanotubes^43^, exhibits corrugations with atomic-scale periodicities. On the other hand, those atomically corrugating features explicitly decline in Fig. 1e and become inconspicuous in Fig. 1f, corresponding to the **R**_**o**_ = 7.5 Å and 6.5 Å nanotubes, respectively.

**Figure 2:**
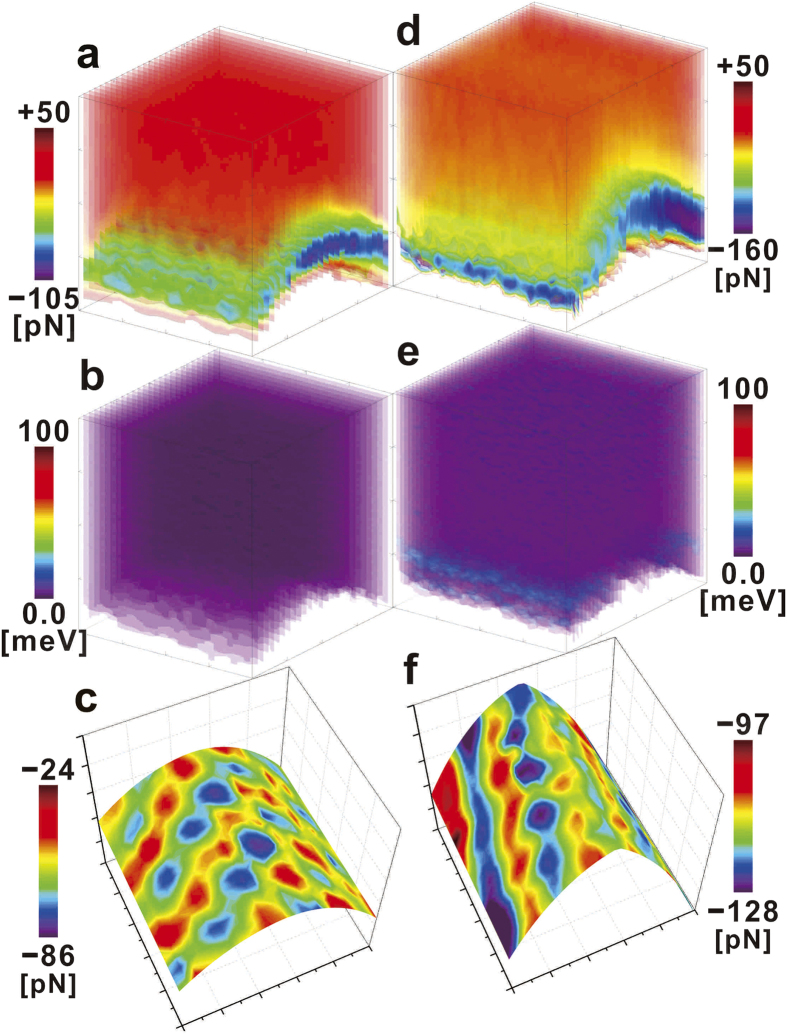
Atomic-scale 3D force and damping spectroscopy on convexly curved graphene of different curvatures. (**a**,**d**) The 3D force fields F(*x, y, z*) obtained over the nanotubes of R_o_ = 8.1 Å (**a**) and 6.5 Å (**d**) within a rectangular parallelepiped (10 × 10 × 6 Å^3^). F(*x, y, z*) was derived from the short-range term of the frequency shift, i.e., Δ*f*_sht_(*x, y, z*), using Sader’s formula^46^,^47^. The Δ*f*_sht_(*x, y, z*) was derived by subtracting the long-range background term from the frequency shift Δ*f*(*x, y, z*) originally obtained when retracting the tip during the measurement^45^,^46^,^47^. (**b**,**e**) The 3D damping fields U_dmp_(*x, y, z*) simultaneously obtained with F(*x, y, z*) in (**a** and **d**), respectively. The locations exhibiting slight amounts of inelastic interactions (10–20 meV) in **e** correspond to the sidewalls of the nanotube with the smaller radius (R_o_ = 6.5 Å), as displayed in (**d**). The locations further apart from the central ridge consist of the steeper sidewalls, in which not only the foremost atom of the tip apex but also its nearest-neighbouring atom was thought to non-elastically interact with the sidewall. (**c**,**f**) The radial force maps F(*x*, θ) corresponding to the convexly curved sections radially crossing the middle of the blue-coloured regions (shaped similar to a “barrel roof”) in (**a** and **d**). F(*x*, θ) almost dependably traces the ridge of the convexly curved graphene, and thereby the curved surfaces in (**c** and **f**) directly represent the differences in their curvatures. The red- and blue-coloured spots in F(*x*, θ) correspond to the carbon atom and hollow sites of the hexagonal honeycomb lattice of the convexly curved graphene, respectively. It should be noted that in F(*x*, θ), the successive distributions of the blue-coloured spots in specific directions might be induced by superposition of the interactions successively acting on the “dimer row” of the tip-apex atoms arranging only in the specific direction, as depicted in Fig. 1a. Hence, by excluding the directions exhibiting those artefacts, the force maps showing atomic arrangements certainly enable us to quantitatively analyse the attractive interactions on the atomic scale.

In 3D force fields **F**(***x**, **y**, **z***)^44^,^45^, the regions in which interatomic attractive forces attain their maximum values, i.e., the blue-coloured areas in Fig. 2a,d, correspond to the ridges of the convexly curved single-layered graphene (i.e., the hollow-tubes’ upper halves), as illustrated in Fig. 1a. Thus, the blue-coloured areas in Fig. 2a,d represent the features unique to their different curvatures. The **F**(***x**, **y**, **z***) over the **R**_**o**_ = 8.1 Å and 6.5 Å nanotubes are displayed, respectively, in Fig. 2a,d within a rectangular parallelepiped (10 × 10 × 6 Å^3^). **F**(***x**, **y**, **z***) represents the spatial distributions of the interatomic forces acting exclusively on the foremost atom of the tip apex over the ridges where the 3D topographies in Fig. 1d,f were obtained. The interatomic forces were derived by subtracting long-range background forces acting comprehensively on the tip apex towards horizontally wide and perpendicularly intersecting sample areas, including steep sidewalls and plane substrates (Fig. 1a,c).

A comparison of the 3D force fields with the 3D damping fields^43^,^45^ simultaneously measured revealed that the foremost atom of the tip apex exclusively contributes to the elastic interactions with the individual carbon (**C**) atoms of the central ridge. The 3D damping fields **U**_**dmp**_(***x**, **y**, **z***) in Fig. 2b,e three-dimensionally specify the locations in which inelastic interactions occurred within the same parallelepiped as shown in Fig. 2a,d, respectively. Indeed, they are almost completely absent (<3 meV) in Fig. 2b and over the shown in Fig. 2e area, except lower peripheral areas, meaning that the interatomic interactions are elastic over the whole ridge and central ridge of the **R**_**o**_ = 8.1 Å and 6.5 Å nanotubes, respectively.

The contrast in the radial force maps was found to be closely related to the corrugation amplitudes in the 3D topographies, where the atomic features were very prominent for the larger **R**_**o**_ = 8.1 Å (Fig. 1d) but inconspicuous for the smaller **R**_**o**_ = 6.5 Å (Fig. 1f). The convexly curved sections radially crossing the middle of the blue-coloured regions in **F**(***x**, **y**, **z***) (Fig. 2a,d) are presented as “radial force maps” **F**(***x***, **θ**) (Fig. 2c,f, respectively). They almost dependably trace the ridge of the convexly curved graphene. The **F**(***x***, **θ**) maps in Fig. 2c,f are rescaled by individual colour codes in which the least upper and greatest lower bounds are, respectively, set to the minimal and maximal forces. The contrast between the red- and blue-coloured spots in **F**(***x***, **θ**), i.e., the difference between the attractive-force minima and maxima, reaches approximately 40pN for **R**_**o**_ = 8.1 Å (Fig. 2c), whereas it is nearly half (~20 pN) for **R**_**o**_ = 6.5 Å (Fig. 2f).

## Correlation of interatomic forces and potentials to the curvatures

**Figure 3:**
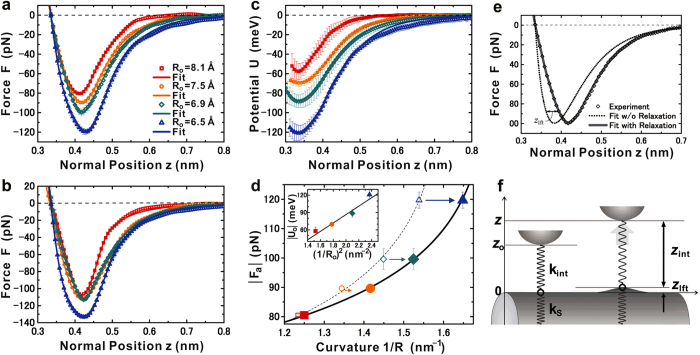
Interatomic force and potential versus normal *z* position at atomically specific sites. (**a**–**c**) Interatomic force **F**(***z***) (**a**,**b**) and potential U(*z*) (**c**) plots and their approximate curves for the carbon (C) atom sites (**a**,**c**) and hollow sites (**b**) in the central ridge of convexly curved graphene with different curvatures. All data plots represent the mean values of the original data sets, corresponding to the C atom sites (**a**,**c**) and hollow sites (**b**) in the topmost part of the nanotubes with different original radii R_o_ = 8.1 Å (red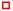), 7.5 Å (orange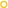), 6.9 Å (green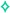), and 6.5 Å (blue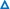). The F(*z*) and U(*z*) were derived from the short-range term of the frequency shift, i.e., Δ*f*_sht_(*z*) using Sader’s formula. Subtracting the “long-range” term from the frequency shift Δ*f*(*z*) originally measured in the 3D spectroscopy yields Δ*f*_sht_(*z*)^46^,^47^. (**d**) The maximum attractive force |F_a_| versus the curvature 1/R of convexly curved graphene. The |F_a_| values were plotted as a function of 1/R, rearranged taking the local relaxation of C atoms into account, indicated by closed large dots, and as a function of the original curvature 1/R_o_, as indicated by open small dots. The solid and dotted curves are the respective approximate curves. Inset: The maximum attractive potential |U_o_| versus the square of the original curvature (1/R_o_)^2^. The individual colours correspond to the variations in curvature, as mentioned above. (**e**) Typical F(***z***) curve. The approximate curve for the data obtained over the standard nanotube of R_o_ = 6.9 ± 0.1 Å was yielded by Eq. (4) in the text, taking the local relaxation of C atoms into account (full line) or not (dotted line). (**f**) Schematic of the coupling mechanism between a tip-apex atom and a carbon atom of convexly curved graphene. The spring constants k_int_ and k_s_ indicate the elastic stiffness of the junction formed by the tip-apex atom and its closest C atom and of the convexly curved graphene at the closest C atom site, respectively. The right and left parts are the cases with and without “lift” displacement *z*_lft_, respectively, which are dependent on the variation in the normal distance *z*_int_ between them.

The red- and blue-coloured spots in Fig. 2c,f, designating the locations of the “relative” minima and the maxima in **F**(***x***, **θ**), can be assigned to the **C**-atom and hollow sites, respectively, because the interatomic attractive force **F**(***z***) acting on the tip-apex atom over the red-coloured spots in **F**(***x***, **θ**) was found to be clearly dependent on the radius **R**_**o**_, whereas the **F**(***z***) curve over the blue-coloured spots showed no clear dependence on **R**_**o**_. The **F**(***z***) at the normal ***z*** position in the out-of-plane direction over the sites corresponding to the red- and blue-coloured spots in **F**(***x***, **θ**) is plotted in Fig. 3a,b, respectively, for the nanotubes of the four different original radii **R**_**o**_ (8.1 to 6.5 Å). The ***z*** position is arranged to be the equilibrium **z**_**o**_(=3.35 Å) position in the case **F**(***z***) = 0. Each plot in Fig. 3a,b is the mean of **F**(***z***) in **F**(***x**, **y**, **z***), respectively, corresponding to the red- and blue-coloured spots around the central ridge in **F**(***x***, **θ**), where the 3D topographies (Fig. 1d–f) exhibit the corrugating features, and **U**_**dmp**_(***x**, **y**, **z***) (Fig. 2b,e) represents the elasticity.

Figure 3a shows that the strength of **F**(***z***) over the red-coloured spots in **F**(***x***, **θ**) is negatively correlated with the original radius **R**_**o**_, that is, positively correlated with the original bending curvature **1**/**R**_**o**_. The positive correlation between **F**(***z***) and **1**/**R**_**o**_ may conflict with the expected negative correlation of “nonbonding” interactions. Over the **C** atoms of graphene, the **π**-orbitals forming a reciprocal weak bond (i.e., **π**-bond) predominantly contribute to the attractive forces acting on the tip-apex atom, unless any electron-transfer reactions occur^5^,^46^,^47^. Since not only the closest atom but also the nearest neighbours additively contribute to such “nonbonding” interactions, following the inverse power law, the latter’s contributions decrease as their distances **d**_**int**_ to the tip-apex atom increase with **1**/**R**_**o**_, as depicted in Fig. 1g. On the other hand, Fig. 3b shows that the **F**(***z***) curves over the blue-coloured spots in **F**(***x***, **θ**) were found to be more independent of **1**/**R**_**o**_. The framework of the hexagonal ring is thought to preserve its original structure even in rather heavily curved graphene. The six individual **C** atoms contribute “nonbonding” interactions equally and are always hexagonally arranged around the hollow site.

The depths of the potential wells of the tip-apex atom over the locations assigned to the **C** atoms show a quadratic relationship to the original bending curvature **1**/**R**_**o**_ of the convexly curved graphene. For **F**(***z***) in Fig. 3a, the mean of the potential **U**(**z**), averaging over the locations corresponding to the red-coloured spots around the central ridge in **F**(***x***, **θ**), is plotted in Fig. 3c such that the normal ***z*** position at the depth of the potential well (i.e., **U**(***z***) = **U**_**o**_) should be the equilibrium ***z***_**o**_(=3.35 Å) position for the nanotubes with the four different radii **R**_**o**_ (8.1 to 6.5 Å), respectively. The well depths |**U**_**o**_| are plotted as a function of the square of the original bending curvature (**1**/**R**_**o**_)^2^ in the inset of Fig. 3d. An approximate straight line showing good agreement with all the data indicates that |**U**_**o**_| (i.e., binding energy) is proportional to the square of **1**/**R**_**o**_. Thus, the inset of Fig. 3d supports the validity of the continuum mechanics theories^6^,^30^,^46^,^47^.

To describe the interatomic potentials of curved graphene, Kostov *et al*. proposed a simple bond parameter of the “mixed” state, consisting of the linear combination of the ***sp***^**2**^ and ***sp***^**3**^ bond states by introducing a curvature parameter, ***g***(**1**/**R**), and using the corresponding bond parameters, 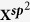 and 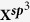, respectively^48^. This method is based on the interatomic potential functions developed for carbon atoms with ***sp***^**2**^ and ***sp***^**3**^ hybridisation and derives new parameters for carbon atoms with **π**-***σ*** re-hybridisation explicitly dependent on the curvature^48^. We adopt this method to describe *U*(***z***) in our study. As such, |**U**_**o**_| can be described as a function of **1**/**R** using the “mixed” state based on the linear combination of the corresponding ***sp***^**2**^ and ***sp***^**3**^ values of the bond parameters 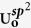 and 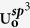, respectively, as follows:


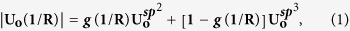


where the curvature parameter ***g***(**1**/**R**) is defined as


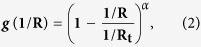


**1/R**_**t**_ is the reference constant and **α** is a positive number. The superior approximation of all four |**U**_**o**_| values was obtained by adopting 
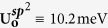
 and 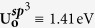
49 in Eq. (1) and by adopting **1/R**_**t**_ ≡ 1.724 nm^−1^ and **α** ≡ 0.027 in Eq. (2), respectively. The former well-depth parameter 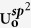 was obtained by adapting the Lennard-Jones parameter for **Ar** atoms^50^ to those for **Si** and **C** atoms, and adapting the latter 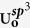 by the **Si-C** binding energy for single-layered graphene^49^. The reference constant **1/R**_**t**_ was based on the radius of curvature **R**_**t**_ = 5.8 Å of the tip apex, estimated by analysing the 3D topographies^51^ in our study.

Figure 3c shows approximate curves to experimental-data plots that were derived from the expansion of Eq. (1), where the Lennard-Jones and Morse potential functions were adopted to describe the bond parameters, 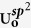 and 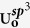, respectively, corresponding to the ***sp***^**2**^ and ***sp***^**3**^ hybridisations, as follows:


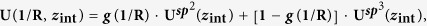



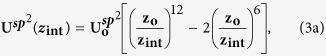



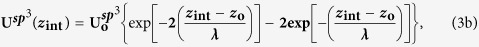


where the decay length parameter ***λ*** in the Morse potential was individually estimated to find an excellent fit to the experimental-data plots. As illustrated in Fig. 3f, the normal position of the **C** atom was set to the origin such that the tip-apex atom is located at the equilibrium ***z***_**o**_(=3.55 Å) position. By adopting the “lift” displacement ***z***_**lft**_ of the **C** atom, corresponding to the relaxation originating from the interatomic attractive forces applied by the tip-apex atom, the interval ***z***_**int**_ between those two atoms is properly described as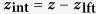. The approximate curves to the force plots in Fig. 3a were obtained by differentiating Eqs (3a) and (3b) in the interval ***z***_**int**_, as follows:


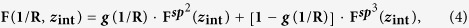



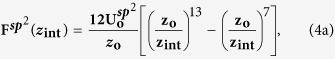



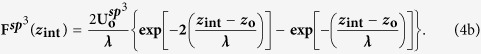


In contrast, differentiating only Eq. (3a) yields the approximate curves to the force plots in Fig. 3b, over the hollow sites, exhibiting no clear dependence on the curvatures.

Since single-layered graphene has an extremely small out-of-plane stiffness^6^,^7^, the closest **C** atom of the convexly curved graphene is expected to be lifted towards the tip-apex atom due to the interatomic attractive force in close proximity, as depicted in Fig. 3f. Consequently, **1/R** would locally increase further from **1/R**_**o**_. The absolute values of the minima in **F**(***x***), i.e., the attractive-force maxima 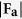, are plotted as a function of **1/R** in Fig. 3d, where the **1/R** values were rearranged taking the “lift” displacement ***z***_**lif**_ into account. The solid curve 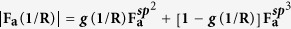 in Fig. 3d was obtained by adopting 
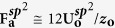
 and 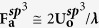. Assuming **1/R** rigidly stays at **1/R**_**o**_ without any relaxation, then the relationship of 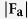 versus **1/R**_**o**_ is additionally given by the plots with open (small) markers and their approximate (dashed) line in Fig. 3d. The force curve estimated under this assumption is represented by the dotted line in Fig. 3e, showing large deviations in the normal ***z*** direction from the experimental-data plots for **R**_**o**_ = 6.9 ± 0.1 Å. In contrast, the approximate curve (solid line), showing excellent agreement with all the experimental-data plots in Fig. 3e, was obtained by rearranging **1/R**. Indeed, the approximate curves in Fig. 3a, showing excellent agreement with all experimental-data plots, were derived from Eq. (4) using the rearranged **1/R** in Eq. (2).

An empirical analysis of the experimental finding of how much **1/R** would locally increase from **1/R**_**o**_ revealed that within the first-order approximation, the local increment of the curvature, i.e., 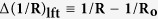, would not be inversely but would be directly proportional to the “lift” displacement ***z***_**lft**_:


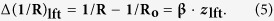


The ***z***_**lft**_ and 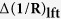 values were estimated in the process of determining the approximate curves to the experimental-data plots in Fig. 3a,c. The resultant positive value **β** is a linear coefficient corresponding to an increasing rate and can be expressed as a linear function of **1/R**_**o**_, as follows:


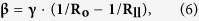


where the linear coefficient **γ** and the lowest limit of the strained curvature 1/**R**_**II**_ were estimated to be 7.770 nm^−1^ and 1.185 nm^−1^, respectively, from the values shown in Table 1.

## Chemical activation and superhardening of curved graphene

**Figure 4:**
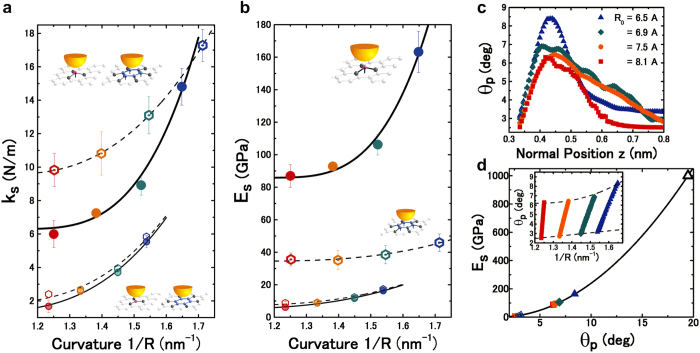
Elastic stiffness and modulus versus graphene curvature and pyramidalization angle. (**a**,**b**) Out-of-plane elastic stiffness k_s_ (**a**) and Young’s modulus E_s_ (**b**) versus the curvature of convexly curved graphene. The closed-circular and open-hexagonal dots (coloured) were, respectively, approximated by the solid and dashed curves expressed by the *n*‒th power of the curvature and obtained at the C atom and hollow sites, respectively, in the topmost part of the nanotubes with different radii R_o_ = 8.1 Å (red), 7.5 Å (orange), 6.9 Å (green), and 6.5 Å (blue). The lower two couples of the dots and curves that almost coincide with each other were obtained in the case where the tip-apex atom was located at the equilibrium *z*_o_ position, as depicted in the bottom corner of (**a**). On the other hand, the upper two couples, showing extremely large discrepancies, were obtained under the maximum “lift” displacement 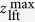, as depicted in the topmost corner of (**a**) and in (**b**). The E_s_ value was derived from the k_s_ value based on the simple atomistic model [Method]. (**c**) The Pyramidalization angle θ_p_ versus the normal *z* position of the tip-apex atom. The individual θ_p_ values were derived from the atomistic model based on the *z*_lft_ of the C atom and its relationship to the local curvature increment Δ(1/R)_lft_ in Eq. (5) (see text). The plots at the left ends and at the maximum points correspond to the original 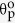 at *z*_o_ (F(*z*_o_) = 0 and *z*_lft_ = 0) and the maximum 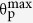 under the maximum 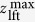, respectively. The variations of decay in the right side beyond the maximum points can be ascribed to two different kinds of behaviour, which most likely depend on the helical indices. (**d**) Out-of-plane Young’s modulus versus pyramidalization angle. The relations of 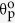 and 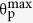 to E_s_ were plotted together with a data point (open triangle) of the tetrahedral bond angle (θ_p_ = 19.5°) for the *sp*^3^ hybridisation in diamond (E_s_ ≅ 1 TPa). Inset: The Pyramidalization angle versus the curvature of convexly curved graphene with four different original curvatures, i.e., the nanotubes with the different above-mentioned radii R_o_.

Figure 4a explicitly shows that the out-of-plane elastic stiffness **k**_**S**_ of the convexly curved graphene [Method] attains a much larger maximal value 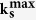 under the maximal “lift” displacement 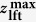 than the original value 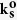 at the equilibrium ***z***_**o**_ position (***z***_**lft**_ = 0). The upper two variations of the plots in Fig. 4a show that 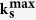 (at 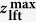) represents conspicuous differences between the **C**-atom and hollow sites in the variations as a function of the maximal-strained curvatures 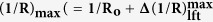). On the other hand, the lower two variations show that the two values for 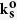 (at ***z***_**lft**_ = 0) almost coincide with each other for all the original bending curvatures **1**/**R**_**o**_. The relationship of 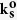 with **1**/**R**_**o**_ can be described by the ***n***-th power of **1**/**R**_**o**_, i.e., 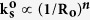, where ***n*** was estimated to be 2.82 and 2.86 for the **C**-atom and hollow sites, respectively. While the relationship of 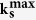 with (**1**/**R**)_**max**_ follows the relationship 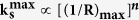 as well, ***n*** was estimated to be slightly lower and much higher at the hollow and **C**-atom sites, at 2.38 and 3.46, respectively.

In the case that the tip-apex atom is located at the equilibrium ***z***_**o**_ position (***z***_**lft**_ = 0), the out-of-plane Young’s modulus 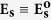, ranging from 7.1 to 13GPa [Method], was found to be independent of any atomically specific site and almost uniform over the whole ridge, following the relationship 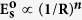, where ***n*** was estimated to be 2.67 and 2.87 at the **C**-atom and hollow sites, respectively. These results show excellent agreement with those of many previous reports on the nanoindentation and compression of carbon nanotubes by the AFM tip, for which the Hertzian model based on the plate idealisation of continuum mechanics is still applicable^52^. On the other hand, the maximal 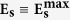 under the maximal “lift” displacement 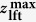 explicitly demonstrates a conspicuous disparity or difference between the **C**-atom and hollow sites, resulting in tremendous atomic-site dependency. The maximal-strained curvature (**1**/**R**)_**max**_ and 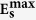 were found to follow the relationship 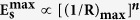, where ***n*** was estimated to be 2.80 at the hollow sites but 3.47 at the **C**-atom sites, as in the case of 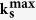. However, more interestingly, the **C**-atom sites exhibit a much more pronounced dependence of 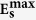 on (**1**/**R**)_**max**_, attaining a significantly large value of 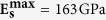, which is almost comparable to that of silicon (i.e., the tip-apex material) with a so-called diamond structure holding ***sp***^**3**^ orbitals in a tetrahedral framework^53^.

To elucidate the reason why **E**_**s**_ is dramatically higher at the **C**-atom sites under the maximal “lift” displacement 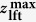, the pyramidalization angle **θ**_**p**_ (see Fig. 1a) was evaluated based on the “lift” displacement ***z***_**lft**_ of the **C** atom and its relationship to the local curvature increment 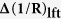. The individual **θ**_**p**_ at the equilibrium ***z***_**o**_ position (***z***_**lft**_ = 0) was first derived for the respective **1**/**R**_**o**_ of the four different nanotubes, taking their helical indices (**n**, **m**) into account. Figure 4c demonstrates the variations of **θ**_**p**_ as a function of the normal ***z*** position of the tip-apex atom, in which the plots at the left ends and the maxima correspond to the original 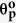 at the equilibrium ***z***_**o**_ position (***z***_**lft**_ = 0) and the maximal 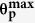 under the maximal 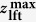, respectively. Furthermore, the plots of 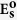 versus 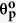 and 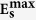 versus 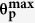 are shown in the bottom and middle groups of in Fig. 4d, respectively, together with a data point (open triangle) for the tetrahedral bond angle (**θ**_**p**_ = 19.5°) in the ***sp***^**3**^ hybridisation of the diamond (**E**_***s***_ ≅ 1TPa), as the upper limit. Interestingly, all the plots were almost on a parabolic line, indicating their quadratic correlation (i.e., **E**_**s**_ ∝ (**θ**_**p**_)^2^).

Furthermore, to gain clear insight into the relationship of **E**_**s**_ versus **1**/**R**, as demonstrated in Fig. 4b, the variations of **θ**_**p**_ were replotted as a function of **1**/**R** in the inset of Fig. 4d. The individual data sets obtained for the four different nanotubes showed that the respective **θ**_**p**_ values linearly increased with 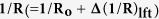. All the data plots in Fig. 4c were aligned into respective rows in the inset of Fig. 4d, and thereby the left-end and the maximum plots, (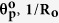) and (
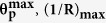
), in Fig. 4c correspond to the lowest and topmost ends of the rows in the inset of Fig. 4d. 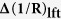 was found to be directly proportional to ***z***_**lft**_, as in Eqs (5) and (6), where the linear coefficient ***β*** also linearly increased with **1**/**R**_**o**_. In contrast, the slope of the rows in the inset of Fig. 4d decreased with **1**/**R**_**o**_. Nevertheless, **θ**_**p**_ attained the maximum 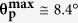 at the maximal-strained curvature (**1**/**R**)_**max**_. The lower approximate straight line, linking (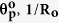) plots, indicated that 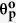 was linearly correlated with **1**/**R**_**o**_. On the other hand, the upper approximate nonlinear line, linking (
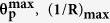
), indicated that 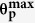 was directly correlated to the ***n***-th power of (**1**/**R**)_**max**_, i.e., 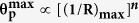, where ***n*** was approximated to be 2.31. The relationships of 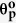 versus **1**/**R**_**o**_ and 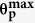 versus (**1**/**R**)_**max**_ corresponding to those of 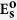 versus **1**/**R**_**o**_ and 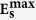 versus (**1**/**R**)_**max**_ in Fig. 4b. Consequently, the dramatic increase in 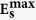 at the **C**-atom sites was found to be closely related to the nonlinear increment of 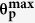, reaching up to 8.4° at the end.

The increase in **θ**_**p**_ would be accompanied by an increase in the out-of-plane attractive potential according to the POAV theory, in which the degree of the valence orbital hybridisation depends on **θ**_**p**_, as illustrated in Fig. 1 23,24,25,26,27: a slight increment in **θ**_**p**_ leads to a continued weak ***π***-state following ***sp***^**2**^ hybridisation, whereas its further increment towards the tetrahedral bond angle (**θ**_**p**_ = 19.5°) yields a transition towards the chemically radical ***σ***-state of a dangling bond following ***sp***^**3**^ hybridisation. Intermingling the ***σ***-state of chemically radical dangling bonds with the nonbonding ***π***-state in the transition from the ***sp***^**2**^ to ***sp***^**3**^ hybridisation (i.e., ***π***-***σ*** re-hybridisation) triggered by the increment in **θ**_**p**_ (3.1° to 8.4°) certainly explains not only the increase in **F**(***z***) but also the significant enhancement of **E**_**s**_ (13 to 163 GPa) specifically at the **C**–atom sites. The 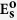 values for the original bending curvatures **1**/**R**_**o**_ were independent of atomically specific sites and almost uniform over the whole ridge, whose correlation to **1**/**R**_**o**_ was covered by continuum mechanics. In contrast, the unexpectedly great variation in 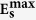 for the maximal-strained curvatures (**1**/**R**)_**max**_, i.e., under the maximal “lift” displacement 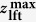 specifically at the *C*-atom sites, could be attributed to the result from the ***π***-***σ*** re-hybridisation, and thereby indicates an atomically pin-point breaking of the continuum mechanics.

## Applicability of our findings

Very recently, the functionalisation of graphene, especially hydrogenation, has attracted much attention for two main reasons: it can be used to tune the band gap for realising semi-conducting behaviour with a high carrier mobility, and it can also be harnessed as an energy-conversion/storage material^7^. For the case of hydrogenation, the ripples with large-curvature, likely narrow or highly curved nanotubes, usually with a diameter <1 nm, have thus far been considered necessary^11^,^12^ for binding hydrogen^54^, leading to the hybridisation of carbon atoms from ***sp***^**2**^ into ***sp***^**3**^, and thereby removing the conducting **π**-bonds and opening an energy gap^54^,^55^,^56^,^57^. However, our findings suggest that the interatomic attractive forces applied by any inactive atom or molecule beyond the tip-apex atom could trigger the transition of its bond state from ***sp***^**2**^ to ***sp***^**3**^ hybridisation, although the original radius of curvature in convexly curved graphene is larger than 5 Å (i.e., >1 nm in diameter). Furthermore, the significant strength enhancement of the out-of-plane elasticity (i.e., superhardening) discovered by our study suggests that the interatomic attractive forces acting between nanostructured graphene and other components would play an important role in enhancing the mechanical strength of composite materials.

## Method

### Out-of-plane elastic stiffness

Using the “lift” displacement ***z***_**lft**_ of the **C** atom, the interatomic forces acting on the tip-apex atom can be expressed as 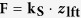 (or 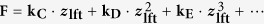) because their interaction was found to be elastic, as demonstrated in **U**_**dmp**_(***x**, **y**, **z***) (Fig. 2b,e). The elastic stiffness **k**_**S**_ of the convexly curved graphene was expected to vary with the local increment of the curvature 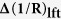, with a linear correlation to ***z***_**lft**_, as described in Eq. (5). Since the applicability of Hooke’s law is guaranteed for the small displacement 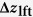, the elastic stiffness **k**_**S**_ can be derived from the infinitesimal force change *d***F** for the infinitesimal displacement *d**z***_**lft**_ as **k**_**S**_ = *d***F**/*d***z**_**lft**_. Indeed, the **k**_**S**_ value was found to be almost constant 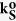 around ***z***_**lft**_ = 0 and attained the maximum 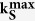 at the maximal “lift” displacement 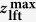. Those 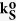 and 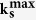 values were plotted as a function of **1**/**R** for the **C**-atoms and hollow sites in Fig. 4a to examine the site dependency on the atomic scale (see illustrations in the inset).

### The out-of-plane Young’s modulus

**E**_**s**_ was evaluated at the **C**-atom and hollow sites based on the simple model, in which the effective areas of interatomic attractive forces applied by the tip-apex atom were estimated taking into account their variations dependent on the “lift” displacement ***z***_**lft**_ of the closest **C** atoms. As illustrated in Fig. 4a,b, the constituent bond elements playing the leading roles are the following: (i) the three in-plane ***σ***-bonds surrounding the closest **C** atom in the case where the tip-apex atom is located directly over it and pulling it up by the maximal “lift” displacement 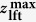; and (ii) the six in-plane ***σ***-bonds surrounding the hexagonal ring in the case where the tip-apex atom is directly over the hollow site and 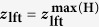. In the case where the tip-apex atom is located at the equilibrium ***z***_**o**_ position (***z***_**lft**_ = 0), where the convexly curved graphene has a curvature of the original **1**/**R**_**o**_ and is free from any local strain, as illustrated in Fig. 3f, the effective areas are expected to be equivalent over the central ridge and directly dependent on **1**/**R**_**o**_, most likely resulting in the variations of 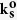 resembling those shown in Fig. 4a. The out-of-plane Young’s modulus **E**_**s**_ can be defined as 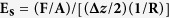, where **A** and **Δz** are the effective area and the out-of-plane displacement, respectively. As plotted in Fig. 4b, the individual **E**_**s**_ values were successfully evaluated at the **C**-atom and hollow (**H**) sites by applying ***z***_**lft**_(=0, 
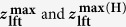
), the corresponding area estimated, and Eq. (5) to **Δz**, **A**, and **1**/**R**, respectively.

## Additional Information

**How to cite this article:** Ashino, M. and Wiesendanger, R. Attractive force-driven superhardening of graphene membrenes as a pin-point breaking of continuum mechanics. *Sci. Rep.* **7**, 46083; doi: 10.1038/srep46083 (2017).

**Publisher's note:** Springer Nature remains neutral with regard to jurisdictional claims in published maps and institutional affiliations.

## Figures and Tables

**Table 1:** The original **1/R**_**o**_ and the increasing rate **β** of the graphene curvature.

R_o_(Å)	8.1 ± 0.3	7.5 ± 0.3	6.9 ± 0.3	6.5 ± 0.3
**1/R**_**o**_(**nm**^**−1**^)	**1**.**236** ± 0.046	**1**.**335** ± 0.053	**1**.**452** ± 0.063	**1**.**542** ± 0.071
**β**(**nm**^**−2**^)	**0**.**4002** ± 0.0017	**1**.**142** ± 0.005	**2**.**021** ± 0.017	**2**.**783** ± 0.004
